# Persistent Synapse Loss Induced by Repetitive LTD in Developing Rat Hippocampal Neurons

**DOI:** 10.1371/journal.pone.0010390

**Published:** 2010-04-28

**Authors:** Yo Shinoda, Tsunehiro Tanaka, Keiko Tominaga-Yoshino, Akihiko Ogura

**Affiliations:** 1 Laboratory for Molecular Neurogenesis, RIKEN Brain Science Institute, Wako, Saitama, Japan; 2 Department of Neuroscience, Graduate School of Frontier Biosciences, Osaka University, Suita, Osaka, Japan; 3 JST-CREST, Kawaguchi, Saitama, Japan; The Research Center of Neurobiology-Neurophysiology of Marseille, France

## Abstract

Synaptic pruning is a physiological event that eliminates excessive or inappropriate synapses to form proper synaptic connections during development of neurons. Appropriate synaptic pruning is required for normal neural development. However, the mechanism of synaptic pruning is not fully understood. Strength of synaptic activity under competitive circumstances is thought to act as a selective force for synaptic pruning. Long-term depression (LTD) is a synaptic plasticity showing persistent decreased synaptic efficacy, which is accompanied by morphological changes of dendritic spines including transient retraction. Repetitive induction of LTD has been shown to cause persistent loss of synapses in mature neurons. Here, we show that multiple, but not single, induction of LTD caused a persistent reduction in the number of dendritic synapses in cultured rat developing hippocampal neurons. When LTD was induced in 14 days *in vitro* cultures by application of (RS)-3,5-dihydroxyphenylglycine (DHPG), a group I metabotropic glutamate receptor (mGluR) agonist, and repeated three times with a one day interval, there was a significant decrease in the number of dendritic synapses. This effect continued up to at least two weeks after the triple LTD induction. The persistent reduction in synapse number occurred in the proximal dendrites, but not the distal dendrites, and was prevented by simultaneous application of the group I/II mGluR antagonist (S)-a-methyl-4-carboxyphenylglycine (MCPG). In conclusion, we found that repetitive LTD induction in developing neurons elicits synaptic pruning and contributes to activity-dependent regulation of synapse number in rat hippocampal neurons.

## Introduction

Synaptic pruning is the morphological loss of excessive synapses during development. In the developmental stage, synaptic pruning refers to the loss of excessive neuronal connections of which experience is thought to be a primary contributor [1]. Synaptic pruning in the early developmental stage is known to occur in many brain regions including the cerebral cortex, cerebellum, olfactory bulb, and hippocampus [2]–[6]. Synaptic pruning is hypothesized to be required for learning and memory [7]. In addition, in computational neurology, synaptic pruning can contribute to memory formation [8]. Abnormal pruning of synapses during development is thought to result in neurodevelopmental disorders such as Rett syndrome [9] and in psychiatric disorders such as schizophrenia [10], [11]. Hence, although appropriate synaptic pruning during development is considered essential for normal brain development [12], the physiological mechanism of the synaptic pruning is not fully understood.

Traditionally, synaptic pruning is considered to depend on synaptic inactivity, or relative inactivity under competitive circumstances. For example, blockade of synaptic activity by AMPA receptor inhibition causes spine pruning in the active synapses between Purkinje cells and climbing fiber synapses [13]. Recently, activity-dependent loss of synapses was also proposed as a mechanism of synaptic pruning [8], [14]–[16]. Moreover, sensory deprivation was shown to prevent dendritic spine loss in the primary somatosensory cortex [17]. Long-term depression (LTD) is the synaptic activity thought to underlie learning and memory [18], [19], which is thought to be accompanied by the remodeling of neural circuit. Although LTD induced synapse elimination was previously demonstrated as the mechanism of structural refinement of synaptic circuit [20]–[22], these synaptic morphological changing is observed only for a few hours. We previously reported that induction of repetitive LTD, but not single LTD, in mature stage cultured hippocampal slices resulted in long-lasting reduction of synaptic transmission accompanied by loss of synapses [23], [24]. Furthermore, repetitive-LTP was reported to induce long-lasting enhancement of synaptic transmission accompanied by creation of novel synapses [25]–[30]. Therefore, we hypothesized that the occurrence of repetitive LTD during development is a physiological mechanism of activity-dependent synaptic pruning. To test this hypothesis, we examined the synaptic pruning caused by repetitive LTD in developing hippocampal neuronal cultures. To induce LTD in cultured hippocampal neurons, we used the group I metabotropic glutamate receptor (mGluR) agonist (RS)-3,5-dihydroxyphenilglycine (DHPG). DHPG activates group I mGluR and can induce LTD in hippocampal slices [23], [31] and cultured hippocampal neurons [32].

Here we show long-lasting pruning of synapses caused by repeated induction of LTD by DHPG treatment in developing cultured hippocampal neurons. This long-lasting pruning was not observed in single DHPG treatment and was blocked by simultaneously application of (S)-a-methyl-4-carboxyphenylglycine (MCPG), a group I/II mGluR antagonist. Furthermore, we found that the long-lasting pruning of synapses showed a different pattern between proximal and distal dendritic regions. This phenomenon is a potential mechanism of synaptic pruning in developing neurons.

## Materials and Methods

### Ethics Statement

Pregnant Wistar/ST rats were housed individually under 12-hour light/12-hour dark cycles for two weeks, in order to synchronize the phase of their internal clocks to the light/dark cycles. All the cages were placed in light-tight cabinets where temperature (23±1°C) and humidity (55±10%) were kept constant. Animals had access to food and water *ad libitum*. We followed the Fundamental Guidelines for Proper Conduct of Animal Experiment and Related Activities in Academic Research Institutions under the jurisdiction of the Ministry of Education, Culture, Sports, Science and Technology, and all of the protocols for animal handling and treatment were reviewed and approved by the Animal Care and Use Committee of Osaka University (#07–032).

### Hippocampal culture and pharmacological treatment

Hippocampal dissociated neurons were prepared according to a partly modified method of that previously described [30]. Embryonic hippocampal neurons were obtained from deeply anesthetized pregnant Wistar/ST rats at 19 days gestation. Individual cells were mechanically isolated by trypsination and trituration. Neurons were plated onto 0.04% polyethyleneimine-coated glass coverslips dropped in 4-well dishes at a density of 5×10^4^ cells/well for immunocytochemistry. Cultures were maintained in B27-supplemented Neurobasal medium (GIBCO-BRL, NY) with 0.5 mM L-glutamine at 37°C in a 95% air/5% CO_2_ humidified atmosphere. For electrophysiology, we used plastic dishes coated with 0.01% poly-L-ornithine at a density of 2×10^6^ cells/100 µl/dish. The cells were incubated in DMEM/F-12 medium (GIBCO-BRL, NY) supplemented with putrescine (0.1 mM), sodium selenite (30 nM), L-glutamine (1.4 mM), gentamicin (10 g/ml), insulin (5 g/ml), and fetal calf serum (10%) at 37°C in a 95% air/5% CO_2_ humidified atmosphere [33]. Half of the medium was replaced once a week with fresh medium. After 14 days *in vitro* (DIV), neurons were incubated for 10 min with 50 µM DHPG to induce LTD, and then washed once with fresh medium. Repetitive induction of LTD was carried out once a day, with a 24 h interval between each application. The group I/II mGluR antagonist MCPG (TOCRIS Bioscience, Bristol, UK; 250 µM) was used 10 min before and during DHPG application. Cell viability before and after pharmacological stimulation was estimated by cell counting in the image taken from the same region of the well at 14, 21, 28, and 35 DIV as described previously [30]. A 225 µm×225 µm area located at approximately the center of vessel was photographed with an Olympus IX-50 inverted epifluorescence microscope with phase-contrast optics. In each of the micrographs, we counted the neuronal number, with the criterion of neuron being one having a phase-bright cell soma.

### Miniature EPSC recording and analysis

Whole-cell recordings from 14–16 DIV pyramidal neurons were obtained using a partly modified method of that previously reported [34]. Cells were voltage clamped at their resting membrane potentials (from −60 to −70 mV) using an intracellular solution, which comprised the following: K-gluconate (116 mM), NaCl (8 mM), HEPES (10 mM), EGTA (0.2 mM), MgCl (2 mM) ATP (2 mM), Na_2_GTP (0.3 mM), and QX-314 (5 mM) adjusted to pH 7.4 with KOH. Cells were perfused continuously with HEPES-buffered saline (HBS) of the following composition: NaCl (140 mM), KCl (3.5 mM), HEPES (10 mM), glucose (20 mM), CaCl_2_ (1.8 mM), MgCl_2_ (0.8 mM), and TTX (0.0001 mM) adjusted to pH 7.4 with NaOH. DHPG was applied by addition to the HBS perfusate excluding TTX. Data were filtered at 2 kHz and digitized at 10 kHz. Continuous recording of miniature EPSCs (mEPSCs) was performed using EPC9 and PULSE software (HEKA Electronik, Lambrecht/Pfalz, Germany). Series resistance was measured intermittently during the mEPSC recording, and recordings were discarded if this varied by more than 20%.

### Immunocytochemistry and synapse counting

Following experimental treatment, cultured neurons were fixed with fixative solution (10 mL 4% paraformaldehyde plus 1.5 mL saturated picric acid) for 10 min at room temperature, following by fixation with methanol for 10 min at −20°C. Cultures were rinsed in PBS and then blocked in PBS with 5% goat serum, 0.1% Triton-X, and 0.05% NaN_3_ for 10 min. Cultures were stained with pre- and post-synaptic marker protein antibodies overnight at 4°C (rabbit-anti-synaptophysin, 1∶500, Santa Cruz Biotechnology, Inc., CA; monoclonal-anti-postsynaptic density 95 [PSD-95], 1∶500, Sigma-Aldrich, MO). Cultures were then rinsed in PBS, incubated in blocking buffer for 10 min, and then exposed to appropriate fluorescent secondary antibodies (1∶500, Molecular Probes, CA). Microscopy was performed with an Olympus BX-50 fluorescent microscope using an UPlanFl 40x 0.75 NA objective (Olympus, Tokyo, Japan). Fluorescence images were collected with a Photometrics PXL™ cooled CCD camera and analyzed using IP-Labs software. Immunofluorescence was analyzed along 20 µm of the proximal (<50 µm from soma) and of the distal (>100 µm) dendrites. Both proximal and distal dendritic regions were selected from the same dendritic branch. Immunoreactive synapses were defined as merged and/or apposed fluorescence pre- and postsynaptic marker discrete points along the dendrite. At least five cells were analyzed per culture, and two to six cultures were analyzed per condition. Separate controls were performed with each experiment. Student's *t*-test and ANOVA followed by post hoc test were used to determine statistical significance.

## Results

### Transient application of DHPG can induce LTD revealed by mEPSCs in cultured rat hippocampal neurons

**Figure 1:**
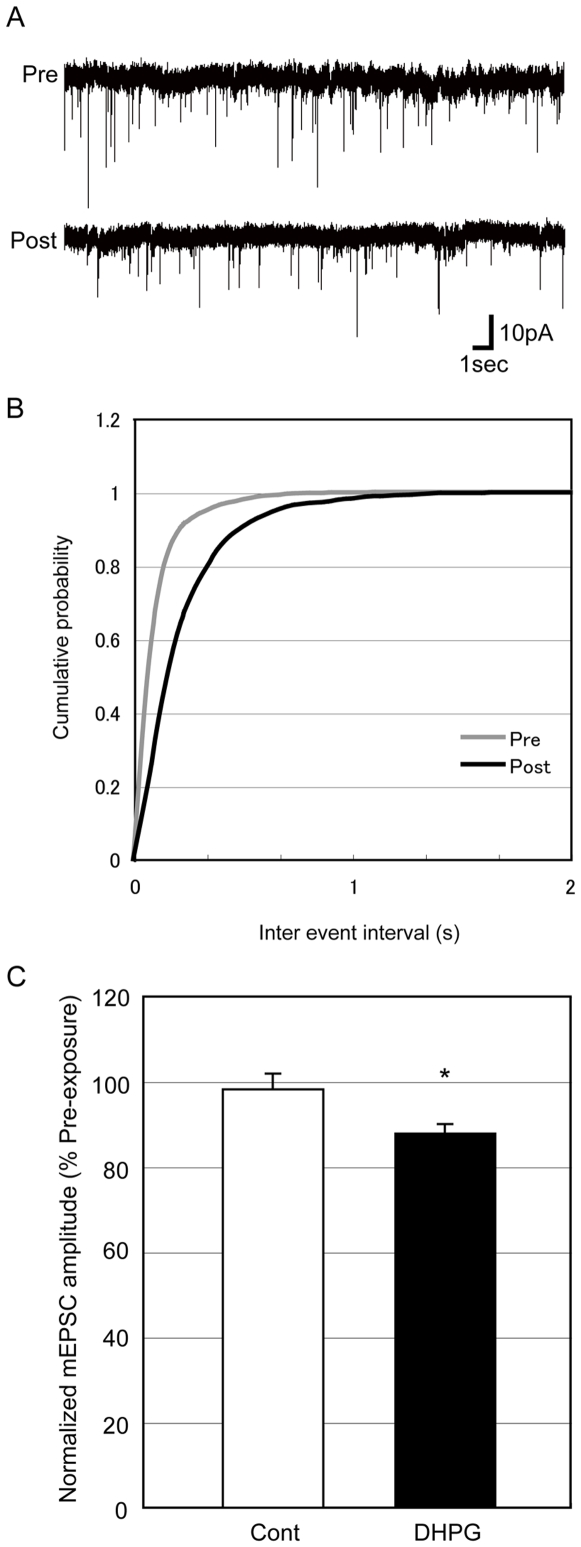
DHPG exposure of cultured hippocampal neurons for 10 min results in LTD of mEPSC amplitude. (A) Representative traces of mEPSCs before (Pre) and 30 min after (Post) exposure to DHPG. (B) Cumulative probability of mEPSC 10-0 min before (Pre) and 20–30 min after (Post) DHPG treatment. *p<0.05 using the Kolmogorov-Smirnov test (n = 9 cells per group) (C) Summary of average change in mEPSC amplitudes. Miniature EPSCs recorded over a 10 min period 30–40 min after DHPG exposure and mock stimulation (Cont) were measured and compared with those collected before exposure (n = 9 cells per group). Error bars indicate SEM; *p<0.05, Student's *t*-test.

To confirm whether DHPG can induce LTD in cultured neurons as reported previously [32], bath application of DHPG was performed under the patch-clamp recording (Fig. 1). Stable recordings of up to 1 h were obtained from 18 neurons of cultured rat hippocampus (nine cells for each of control and DHPG treatment groups). LTD was not associated with any change in holding current or access resistance (data not shown). The mEPSC frequency during 20–30 min after DHPG treatment was reduced comparing with the frequency during 10-0 min before DHPG treatment (Fig. 1B: tau [ms]  = 98.4 and 252.2 in Pre and Post, respectively). The mEPSC amplitude at 30 min after DHPG exposure was reduced to 88% of the amplitude before exposure (Fig. 1C).

### Repetitive LTD induction does not alter cell survival

**Figure 2:**
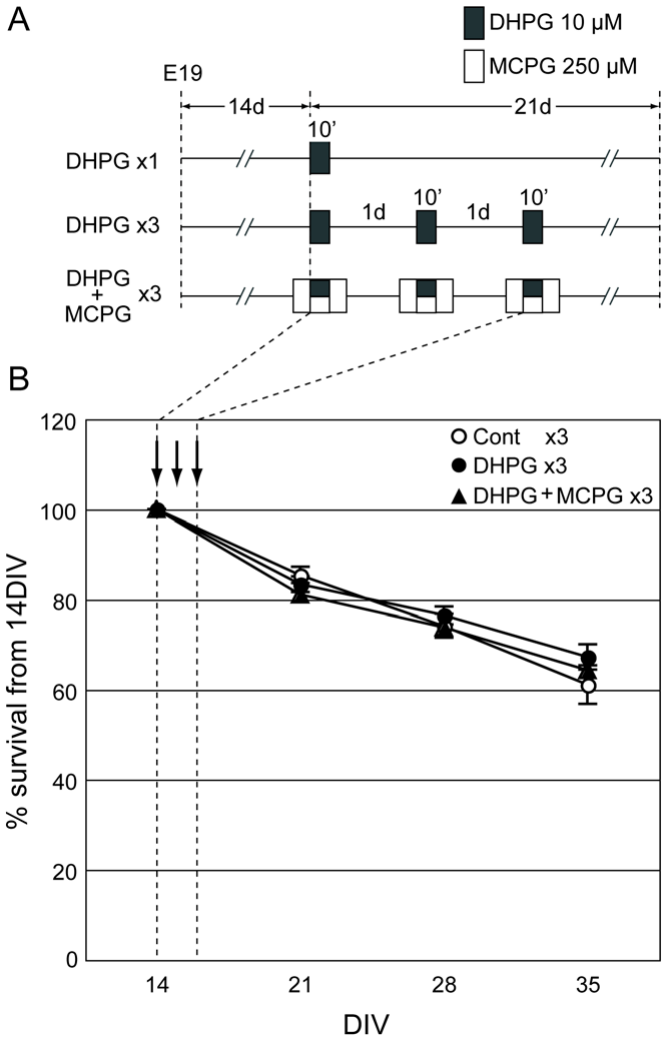
Drug application and cell viability. (A) Cultured neurons at 14 DIV were treated with DHPG (filled boxes, 10 µM, 10 min) with or without MCPG (open boxes, 250 µM, 30 min). Drugs were applied once a day, either singly or three times. MCPG was applied from 10 min prior to 10 min after DHPG exposure. (B) The survival rate during the experimental period. Arrows indicate the days of drug application. The number of surviving cells was slightly decreased after repetitive control (open circle), DHPG (filled circle), and DHPG with MCPG (filled triangle) treatment, although there was no significant difference between the groups. The numbers of cultures for each plot were 11 (control), 12 (DHPG), and 6 (DHPG+MCPG), respectively. Error bars indicate SEM.

The schematic protocol of LTD induction during cell culture is shown in Figure 2A. We adopted a protocol of one versus three applications of DHPG to induce single and repetitive LTD, respectively. MCPG was used to prevent the DHPG-induced LTD. As the cell survival rates may be affected by drug treatments, we compared the cell viability of all protocols. Results showed no change in cell survival rates in any protocols (Fig. 2B), suggesting that the cell viability was not altered by DHPG or MCPG treatment.

### Proximal but not distal dendritic synapses were lost at a few days after repetitive LTD induction

**Figure 3:**
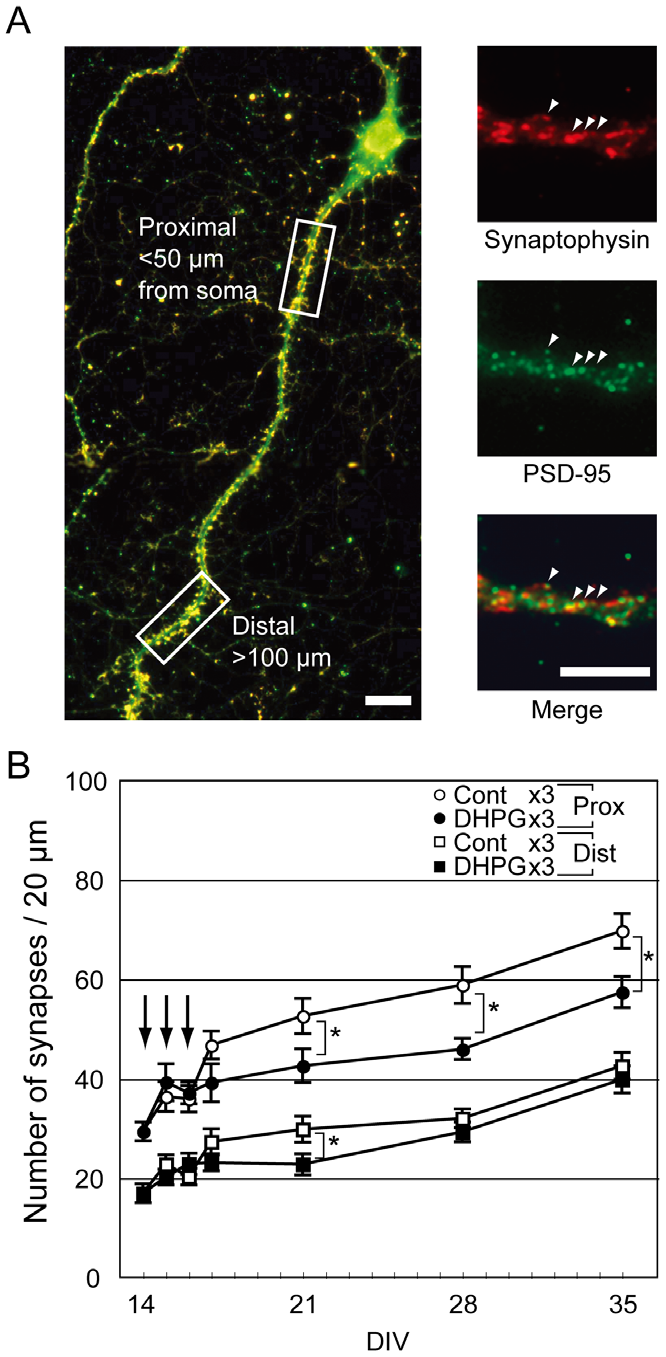
Time-dependent changes of synapse number after drug treatments. (A) Representative images of synaptophysin (red) and PSD-95 (green) immunostaining. The continual dendrite of a pyramidal neuron was divided into proximal and distal regions as determined by distance from soma (<50 µm and >100 µm, respectively). Two boxes indicate 20 µm regions of interest. Merged and apposed points of synaptophysin and PSD-95 immunopositive puncta were regarded as synapses (arrowheads). Scale bars indicate 10 µm. (B) Time-dependent changes of synapse number. Arrows indicate the days of drug application. In the proximal dendritic region, three applications of DHPG (filled circle) significantly reduced the numbers of developing synapses from one week after first drug application compared with control (open circle). In the distal region, although there was a significant difference between control (open square) and DHPG (filled square) groups at one week after first treatment, there was no persistent difference in number of synapses. The numbers of each experiment were indicated on top of each bar at DIV 14, 21, 28, and 35, respectively in all groups. Error bars indicate SEM; *p<0.05, Student's *t*-test.

We used antibodies against pre- and post-synaptic marker proteins synaptophysin and PSD–95 to determine the synaptic sites. Both pre- and post-synaptic puncta were clearly identified in all culture conditions (Fig. 3A). We chose neurons that had a long traceable dendrite containing a 20 µm region of interest that did not cross or extend along any other dendrites (Fig. 3A). Proximal and distal dendritic regions were determined using the dendritic longitudinal length from the soma. In most cases, as the dendritic thickness in the proximal dendrite was larger than in the distal dendrite (data not shown), the number of synapses in the proximal dendrite was also larger than the distal dendrite (Fig. 3B). The number of synapses in both dendritic regions was increased during the experimental period in both control and DHPG treatment cultures. However, the number of synapses in the proximal dendrite was reduced to low levels by repetitive LTD induction when compared with repetitive control stimulation (Fig. 3B). This synaptic reduction occurred immediately after the third DHPG stimulation and the range of the reduction was maintained over two weeks. In the distal dendritic region, a transient difference in synapse number was observed at one week after the first treatment, although there was no difference after the following week (Fig. 3B).

### Synaptic reduction is generated by LTD repetition and prevented by blockade of LTD induction

**Figure 4:**
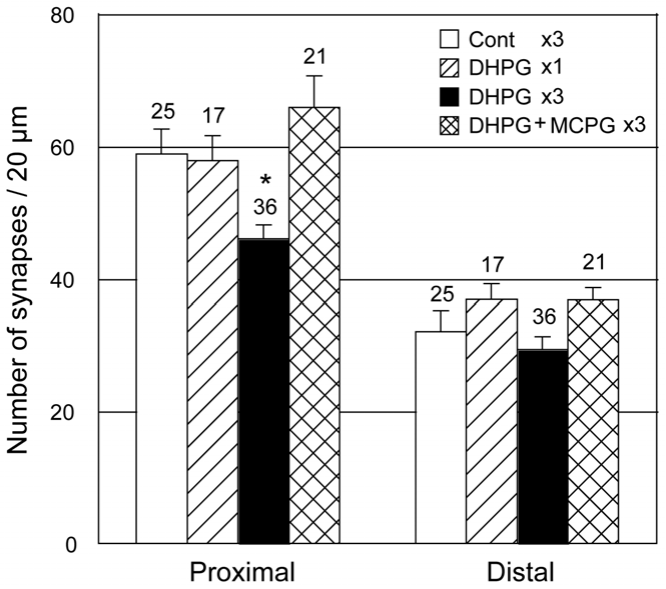
Repetitive LTD-dependence of long-lasting synaptic reduction. Single (diagonal bar) and three times (filled bar) application of DHPG were compared with control (open bar) at 14 days after first drug treatment. MCPG applied with DHPG (hatched bar) prevented long-lasting synaptic reduction compared with DHPG treatment. Note that three LTD inductions, but not single LTD induction, generated long-lasting synaptic reduction in the proximal dendritic region. The numbers of each experiment are shown on top of bars. Error bars indicate SEM; *p<0.05, ANOVA followed by post hoc test.

To verify the requirement for repetitive LTD in mediating the decreased numbers of synapses in cultured neurons, we compared synapse numbers at 14 DIV after single versus three times LTD induction (Fig. 4). Similar to previous reports [23], [24], long-lasting synaptic reduction in the proximal dendritic region required three repetitive inductions of LTD; single LTD induction did not generate long-lasting synaptic loss. We used the mGluR agonist DHPG to induce LTD in cultured neurons. As such, blockade of mGluR may prevent the long-lasting synaptic reduction. Simultaneous application of MCPG, a mGluR antagonist, with DHPG application prevented the long-lasting synaptic reduction (Fig. 4). MCPG slightly increased the number of synapses in both proximal and distal dendritic regions rather than preventing the synaptic reduction; however, the differences of synapse number between the control and the MCPG treatment group were not significant (Fig. 4).

## Discussion

We demonstrated that repetitive application of DHPG, which can induce LTD in cultured hippocampal neurons following single exposure, caused a persistent decrease in numbers of synapses. It is already reported that the short-term synaptic elimination induced by LTD [20]–[22]. However the long-lasting decrease in synapse number in the present study required three LTD inductions, as previously reported [23], [24]. Moreover, this decrease occurred only in the proximal dendritic region. We previously reported that repetitive mGluR activation using 1-aminocyclopentane-1, 3-dicarboxylate (ACPD), a group I/II mGluR agonist, caused persistent reduction in synaptic strength in rat hippocampal slice cultures [24]. The long-lasting reduction of synaptic transmission efficiency was accompanied by pre- and post-synaptic morphological deprivation. More recently, we also reported that the long-lasting synaptic reduction could be induced by a repetitive exposure to a variety of chemicals used to induce LTD, including DHPG, N-methyl-D-aspartate (NMDA), and dihydroouabain (DHO) [23]. In the present study, LTD-repetition also induced long-lasting synaptic reduction in cultured developing hippocampal neurons. This synaptic reduction in developing neurons is similar to previous reports with respect to its persistence and dependence on repetition, as well as its independence to cell death.

Activity-dependent synaptic pruning is thought to be dependent on synaptic experience. In the visual cortex, monocular deprivation during a critical period induces homosynaptic LTD [35], [36] and causes drastic spine pruning [6]. In addition, the effect of monocular deprivation occludes subsequent induction of LTD [37]. These data strongly suggest that synaptic pruning in the visual cortex is required for LTD induction. In the monkey prefrontal cortex, the density of excitatory synapses decreases by 40–50% during adolescence [38]. The decrease in synaptic numbers during development occurs in the relatively immature synapses, including those with a lower alpha-amino-3-hydroxy-5-methyl-4-isoxazole propionic acid (AMPA) receptor/NMDA receptor ratio [39]. During LTD, the AMPA receptor is internalized from the surface of the synapse by endocytosis [40] resulting in a reduction in the synaptic AMPA/NMDA ratio [41]. Therefore, LTD may reduce synaptic efficacy via AMPA receptor internalization and subsequent pruning of weaken synapses during development. However, in the present study, induction of repetitive LTD, but not single LTD, was required for long-lasting decreasing of the synapse number. Thus, we suggest that single LTD is used for temporal weakening of synaptic strength, and that synaptic transmission is recovered after a few hours. By contrast, multiple LTD is used for long-lasting synaptic loss (synaptic pruning) to shutout other synaptic transmission.

Most neurons have long dendritic branches, and their physiological features such as protein distribution, electrical conductance and origin of synaptic input are quite different along the longitudinal dendrite. In the present study, the synaptic reduction was observed specifically in the proximal dendritic region, despite the global application of DHPG. A potential mechanism of this region-specific reduction in synaptic expression may relate to differences in receptor distribution. In the adult rat, there are no differences in distribution of mGluR1 and 5 along the dendrites of hippocampal CA1 pyramidal neurons [42]. However, in the newborn (P8) rat, mGluR1a is only expressed in the proximal dendritic region [43]. We used embryonic rat hippocampal neurons and cultured them for a few weeks, thus the developmental stage of the cells may be similar to hippocampal neurons of a newborn rat. Although mGluR5 is a major group I mGluR in rat hippocampal neurons [42], [44], mGluR1 is the main receptor that regulates intracellular calcium accumulation in hippocampal neurons [45]. Furthermore, DHPG-induced LTD requires both mGluR5 and mGluR1 activation [46]. As such, differences in mGluR1 distribution may contribute to the differences in the range of LTD induction along dendrites, which in turn, may regulate the region of synaptic reduction. Differences in the distribution of other receptors may also affect the region of DHPG-induced LTD and synaptic reduction. There is some evidence that mGluR5 physically interacts with the NMDA receptor via scaffolding proteins such as Homer, Shank, and PSD-95 [47]–[49]. The expression of the NMDA receptor subtypes NR1 and NR2A/2B are greater in the soma and the proximal dendritic region compared with the distal dendritic region [50], [51]. An alternative mechanism of region-specific synaptic reduction relates to differences in morphological and electrophysiological features of the membrane along the dendrite. In hippocampal CA1 pyramidal neurons, the number of perforated synapses is smaller in the proximal dendrite [52]. In addition, generally, the dendritic trunk is tapered gradually from the proximal to the distal dendrite, and the distribution of various voltage-dependent channels exhibit regional-specific differences [53]. These features can affect dendritic conductance, which can affect the membrane potential during stimulation [54]. As the membrane potential directly regulates the open probability of NMDA receptors and voltage-gated calcium channels, the morphological and electrophysiological properties of the dendrite are closely related to the intracellular calcium concentration, and may also affect the regional specificity of LTD induction and the generation of the long-lasting synaptic reduction.

In summary, repetitive LTD-dependent long-lasting synaptic loss was observed in developing hippocampal neurons, similar to that previously reported in mature neurons. In addition, the synaptic reduction induced by LTD repetition was only observed in the proximal dendritic region. Further studies are required to clarify the mechanism of activity-dependent synaptic pruning, of which repetitive LTD may be an important component.
